# Dr. Daniel E. Lake

**Published:** 1913-05

**Authors:** 


Dr. Daniel E. Lake, Ecclectric of Philadelphia, 1871, died in Fulton, Oswego County, New York, March 1, aged 75.

